# Genome-wide identification of Aux/IAA and ARF gene families reveal their potential roles in flower opening of *Dendrobium officinale*

**DOI:** 10.1186/s12864-023-09263-y

**Published:** 2023-04-13

**Authors:** Can Si, Danqi Zeng, Jaime A. Teixeira da Silva, Shengxiang Qiu, Jun Duan, Song Bai, Chunmei He

**Affiliations:** 1 Key Laboratory of South China Agricultural Plant Molecular Analysis and Genetic Improvement & Guangdong Provincial Key Laboratory of Applied Botany, South China Botanical Garden, Chinese Academy of Sciences, Guangzhou, 510650 China; 2 South China National Botanical Garden, Guangzhou, 510650 China; 3 University of the Chinese Academy of Sciences, Beijing, 100049 China; 4 Miki-Cho, Kagawa-Ken, Japan; 5 Rice Research Institute, Guangdong Academy of Agricultural Sciences & Guangdong Key Laboratory of New Technology in Rice Breeding & Guangdong Rice Engineering Laboratory, Guangzhou, 510640 China

**Keywords:** *Dendrobium officinale*, *Aux/IAA* genes, *ARF* genes, *Cis*-regulatory element, Flower development

## Abstract

**Background:**

The auxin indole-3-acetic acid (IAA) is a vital phytohormone that influences plant growth and development. Our previous work showed that IAA content decreased during flower development in the medicinally important orchid *Dendrobium officinale*, while Aux/IAA genes were downregulated. However, little information about auxin-responsive genes and their roles in *D. officinale* flower development exists.

**Results:**

This study validated 14 *DoIAA* and 26 *DoARF* early auxin-responsive genes in the *D. officinale* genome. A phylogenetic analysis classified the *DoIAA* genes into two subgroups. An analysis of *cis*-regulatory elements indicated that they were related by phytohormones and abiotic stresses. Gene expression profiles were tissue-specific. Most *DoIAA* genes (except for *DoIAA7*) were sensitive to IAA (10 μmol/L) and were downregulated during flower development. Four DoIAA proteins (DoIAA1, DoIAA6, DoIAA10 and DoIAA13) were mainly localized in the nucleus. A yeast two-hybrid assay showed that these four DoIAA proteins interacted with three DoARF proteins (DoARF2, DoARF17, DoARF23).

**Conclusions:**

The structure and molecular functions of early auxin-responsive genes in *D. officinale* were investigated. The DoIAA-DoARF interaction may play an important role in flower development via the auxin signaling pathway.

**Supplementary Information:**

The online version contains supplementary material available at 10.1186/s12864-023-09263-y.

## Background

Plants produce a variety of primary metabolites that are required for growth and development in the vegetative stage [1]. The growth, productivity and quality of plants are negatively impacted when they are exposed to biotic and abiotic stresses [2]. In medicinal and aromatic plants (MAPs), secondary metabolites (SMs) accumulate in response to these stresses [3]. SMs in MAPs, including polysaccharides, flavonoids, alkaloids, and terpenoids, provide plants with a protective layer, allowing them to survive such stresses, but they also provide multiple benefits to human health via their biological activities, and are thus widely employed in the food, pharmaceutical, and cosmetic industries [4, 5].

SMs are particularly prevalent in *Dendrobium*, a medicinal and ornamental orchid genus [6], and are thus widely used in traditional Chinese medicine [7]. There is a balance between plant yield and SMs in many MAPs such that, under stressful conditions, the growth of MAPs decreases while the production of SMs increases [4]. Previous research focused on the growth, yield and medicinal aspects of MAPs [8]. Despite their important medicinal value, the yield of MAPs, as assessed by plant height, leaf number, stem diameter, fresh weight, and other growth-related parameters, increased at the vegetative stage, and was directed by a range of plant growth regulators (PGRs), including phytohormones [4, 9].

Indole-3-acetic acid (IAA) is an auxin (Aux) that influences cellular and sub-cellular processes, including those related to growth (e.g., cell division, extension and differentiation) and development (e.g., shoot elongation, flower development, and fruit ripening) [10]. Auxins are widely used in plant tissue culture to induce root formation [11]. The auxin-early response gene family, consisting mainly of *Aux/IAA*, *auxin response factor* (*ARF*), *small auxin upregulated RNA* (*SAUR*) and *auxin-responsive gretchen hagen3* (*GH3*) gene families, are involved in the auxin signaling pathway [10]. IAA usually binds to the C-terminal protein–protein interaction domain (CTD) of ARF proteins and activates or represses the function of ARF proteins when plants are exposed to a high or low concentrations of auxin [12]. The B3-type DNA-binding domain (DBD) of ARF proteins binds to the auxin response elements (AuxREs) of auxin-responsive genes and regulates downstream auxin-regulated genes [13].

Auxins modulate cell proliferation and cell expansion in normal organs, and play a critical role in flower development [14]. Auxin induced the constitutive opening of *Nymphaeales* flowers and was involved in floral movement [15]. *SlMBP21*, a MADS-box gene involved in the ethylene and auxin pathways, negatively controlled the size of *Solanum lycopersicum* sepals [14]*.* In *Rosa hybrida*, down-regulation of *RhIAA14* by virus-induced gene silencing reduced cell expansion, leading to reduced petal expansion and thus smaller petals [16]. Moreover, the silencing of *RhIAA16* promoted petal abscission [17].

*Dendrobium officinale* (known as *tie-pi-shi-hu* (铁皮石斛, Tiě Pí Shí Hú) in Chinese) is an important ornamental and medicinal plant of the Orchidaceae. It is listed as a top-grade non-toxic herb in China, and has been widely used in traditional Chinese medicine for thousands of years [18]. Its main medicinal ingredients include water-soluble polysaccharides, flavonoids, alkaloids, and bibenzyls [6]. *D. officinale* is widely cultivated in China and in other Asian countries, and had a market value that exceeded 10 billion RMB (about US$ 1.43 billion) a few years ago [19]. A previous study indicated that IAA content decreased during flower development, between flower bud and fully-opened flowering stages [20]. Despite its cultural and biological importance, few studies have assessed details about the impact of auxin, in specific IAA, and the auxin signaling pathway, on the growth and development of *D. officinale*, especially flower development.

This study aimed to identify the *D. officinale* early auxin-responsive genes in the auxin signaling pathway, as well as *Aux/IAA* and *ARF* gene family members. This information about the *DoIAA* and *DoARF* genes in *D. officinale* will allow plant scientists to better understand the auxin-mediated signaling pathway in flower development, and deepen their understanding of the regulation of orchid plant growth and flowering time.

## Results

### Identification and characterization of *Aux/IAA* and *ARF* genes in *D. officinale*

A total of 14 *Aux/IAA* genes (*DoIAA1*-*14*), were identified in the *D. officinale* genome. The main characteristics of these genes, including their number of amino acids (AA), opening reading frame (ORF) length, protein length, molecular weight (MW), isoelectric point (pI) and prediction of subcellular localization, are listed in Table 1. The length of the ORFs of the 14 *DoIAA* genes ranged from 375 bp (*DoIAA14*) to 918 bp (*DoIAA8*) and the AA length of their respective proteins ranged from 124 to 305 aa. The predicted pI ranged from 5.13 (*DoIAA13*) to 9.36 (*DoIAA2*) and MW varied from 14.21 kDa (*DoIAA14*) to 33.01 kDa (*DoIAA8*). The latter was consistent with ORF length. The results of the prediction of subcellular localization indicated that most of the putative DoIAAs (9 out of 14) were nucleus-targeted proteins, although a few were localized to the chloroplast, cytoplasm and Golgi bodies. A total of 26 potential *ARF* genes were identified in the *D. officinale* genome, and these were named as *DoARF1*-*26*. Detailed information about the 26 *DoARF* genes, including the parameters described above, can be found in Table S1.

**Table 1:** Physiochemical parameters of the 14 DoIAA proteins

Gene ID	Locus	ORF (bp)	AA (aa)	pI	Mw (KDa)	Localization
*DoIAA1*	Dca002725	804	267	5.19	28.48	Nucleus
*DoIAA2*	Dca004451	495	164	9.36	18.99	Chloroplast
*DoIAA3*	Dca005951	531	176	5.68	19.34	Chloroplast
*DoIAA4*	Dca006511	843	280	5.25	31.07	Nucleus
*DoIAA5*	Dca006971	891	296	7.78	32.17	Nucleus
*DoIAA6*	Dca007271	903	300	5.25	32.25	Chloroplast
*DoIAA7*	Dca008472	876	291	8.70	30.95	Nucleus
*DoIAA8*	Dca010891	918	305	6.15	33.01	Golgi
*DoIAA9*	Dca011244	819	272	8.96	29.76	Nucleus
*DoIAA10*	Dca014796	861	286	8.16	30.81	Nucleus
*DoIAA11*	Dca019706	537	178	6.97	20.01	Cytoplasm
*DoIAA12*	Dca024309	453	150	5.14	16.74	Nucleus
*DoIAA13*	Dca026718	459	152	5.13	17.30	Nucleus
*DoIAA14*	Dca027623	375	124	8.69	14.21	Nucleus

### Phylogenetic classification, and conserved domain analysis

**Fig. 1:**
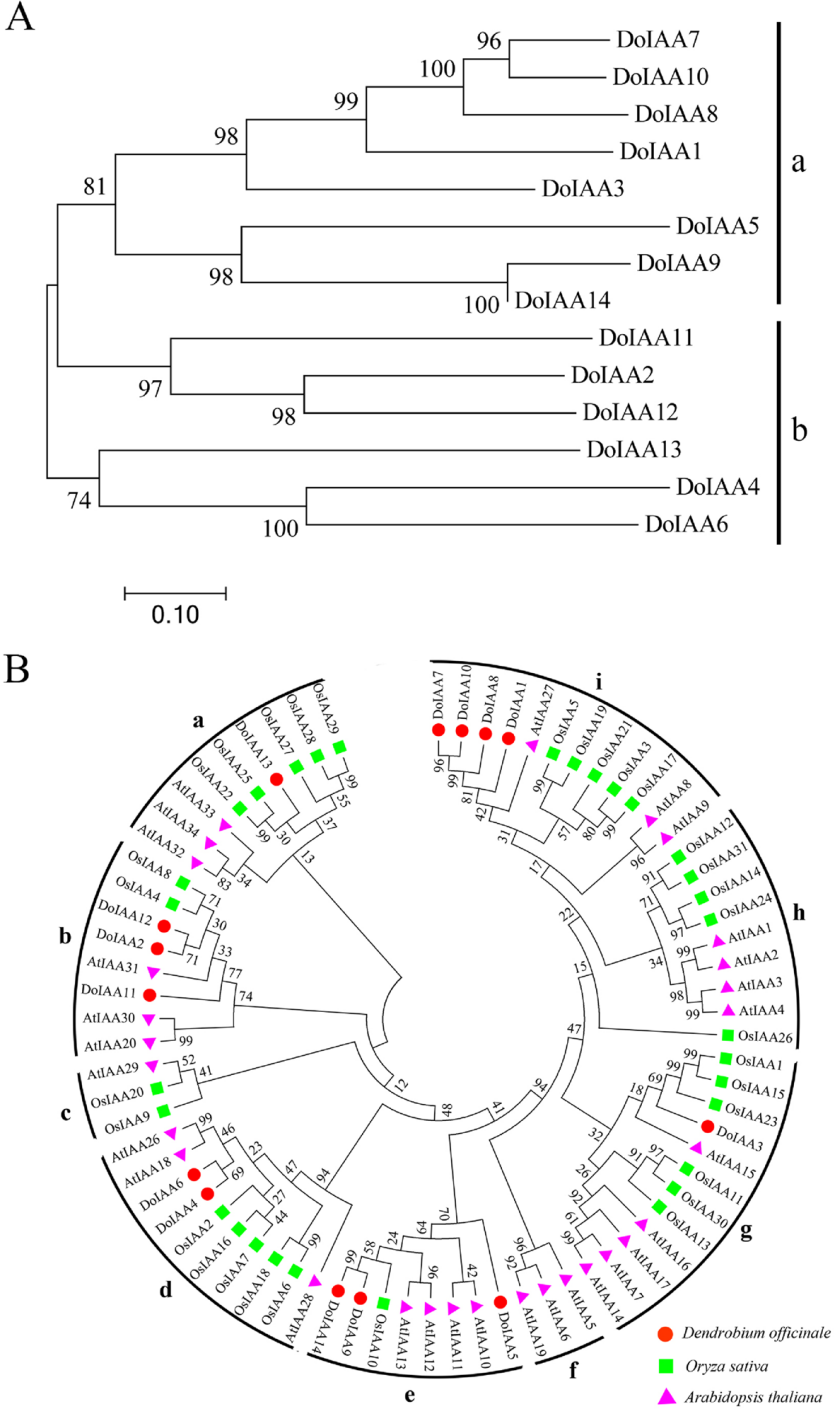
Phylogenetic tree of Aux/IAA proteins from *Dendrobium officinale*, *Arabidopsis thaliana* and *Oryza sativa*. **A** Phylogenetic tree of the predicted Aux/IAA proteins from *D. officinale*. **B** Phylogenetic relationship of Aux/IAA proteins among the three plant species. Red circles, *D. officinale*; luminous green squares, *O. sativa*; pink triangles, *A. thaliana*

The 14 DoIAA proteins were divided into two main groups, a and b (Fig. 1A). Group a was further divided into two subgroups containing five and three members. Group b was also divided into two smaller subgroups, each including three members.

To investigate the phylogenetic relationship among the Aux/IAA proteins, a total of 74 proteins from three plant species, including 14 DoIAA proteins from *D. officinale*, 29 AtIAA proteins from *A. thaliana* and 31 OsIAA proteins from *O. sativa*, were compared, and their AA sequences were used to build a phylogenetic tree (Fig. 1B). The 74 proteins were divided into nine groups, a-i. Two larger groups, g and i, each contained 12 proteins from the three species. The two smaller groups, c and f, included three *A. thaliana* and *O. sativa* proteins, but none from *D. officinale*.

**Fig. 2:**
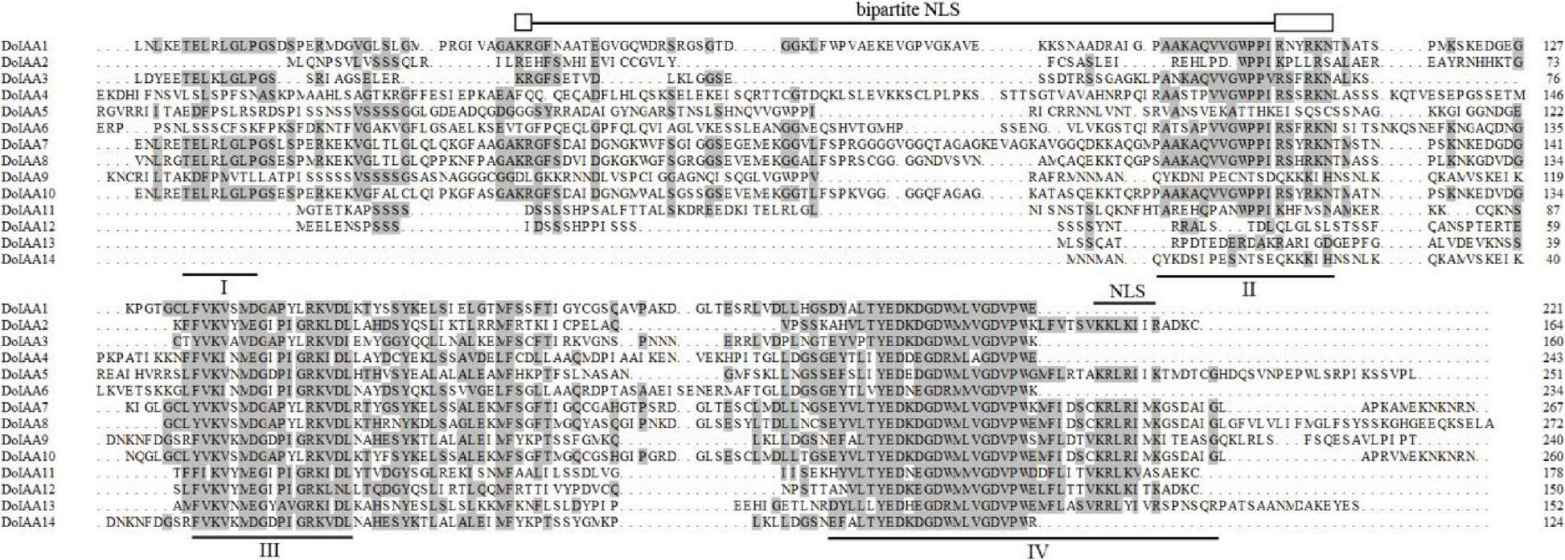
Multiple alignment of 14 Aux/IAA proteins from *Dendrobium officinale*. Conserved domains I, II, III, IV are underlined. Bipartite NLS (between domains I and II), as well as NLS in domain IV, are indicated with a line above them

Multiple sequence alignment revealed that all of the *D. officinale* Aux/IAA proteins displayed over 35% homology, and all harbored four highly conserved domains, which were named I, II, III, and IV (Fig. 2). All of the Aux/IAA proteins carried domains III and IV, but several lacked domains I and/or II. Domain I was absent in DoIAA2, 11, 12, 13 and 14, while domain II was absent in DoIAA5, 9, 12, 13 and 14. Most of the Aux/IAA proteins contained two nuclear localization signals (NLSs), a bipartite NLS and a typical NLS, which were located in domains II and IV, suggesting that these Aux/IAA proteins may be localized in the nucleus (Fig. 2).

### *Cis*-elements analysis of the promoter region

Various types of *cis*-regulatory elements (CREs) related to plant growth and development, and responses to light, phytohormones, stresses and other signals, exist in the promoter regions of *DoIAA* genes. More detailed information about the CREs related to stress and phytohormone responses is available in Figure S1. Five hormone-responsive elements, including to methyl jasmonate (MeJA), abscisic acid (ABA), gibberellin acid (GA), auxin, and salicylic acid (SA), were discovered. Stress-responsive elements, including to anaerobic induction, low temperature and drought, were also detected. These data imply that *DoIAA* genes participate in hormone signaling and stress responses in *D. officinale*.

### Expression patterns of *Aux/IAA* and *ARF* genes

**Fig. 3:**
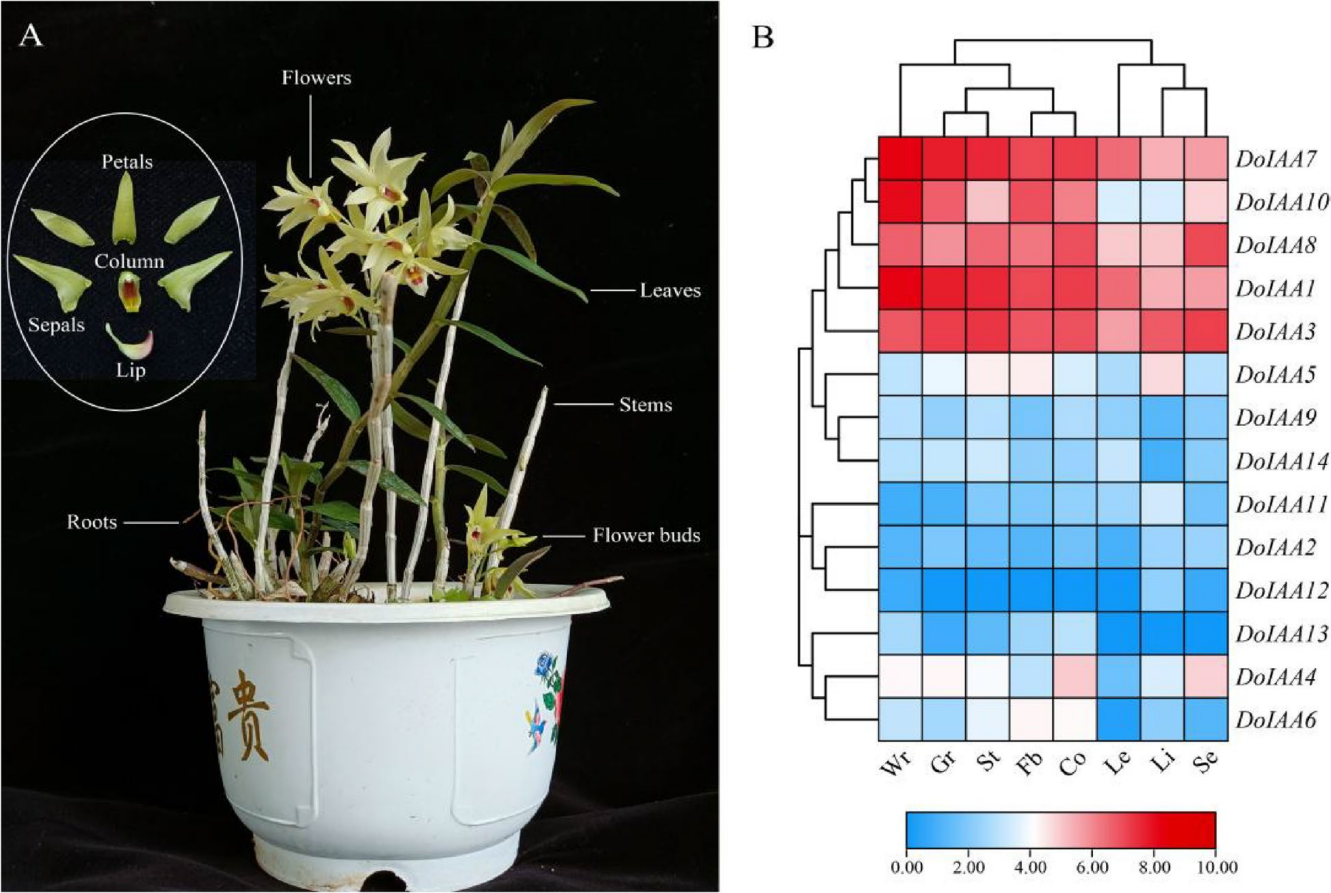
Expression patterns of 14 *DoIAA* genes in different organs of *Dendrobium officinale*. **A** Different organs of *D. officinale*. **B** Transcript profiles of *DoIAA* genes in eight organs of *D. officinale*. Red and blue indicate high and low expression levels, respectively. Wr, white part of roots; Gr, green part of roots; St, stems; Fb, flower buds; Co, column; Le, leaves; Li, lip; Se, sepals

To understand the level of organ-specific expression of the *Aux/IAA* genes, the RNA-seq data of eight tissues of *D. officinale* were analyzed (Fig. 3). The 14 *Aux/IAA* genes displayed an organ-specific expression profile. Five *Aux/IAA* genes (*DoIAA1*, *3*, *7*, *8*, *10*) exhibited relatively higher mRNA levels than other *DoIAA* genes in all eight organs. Three *Aux/IAA* genes (*DoIAA1*, *7*, *10*) displayed a higher expression in the white/green part of roots, indicating their role in root development, while four other *Aux/IAA* genes (*DoIAA4*, *5*, *6*, *8*) were highly expressed in flowers, including the lip, column, sepals and flower buds, suggesting their role in flower development (Fig. 3).

**Fig. 4:**
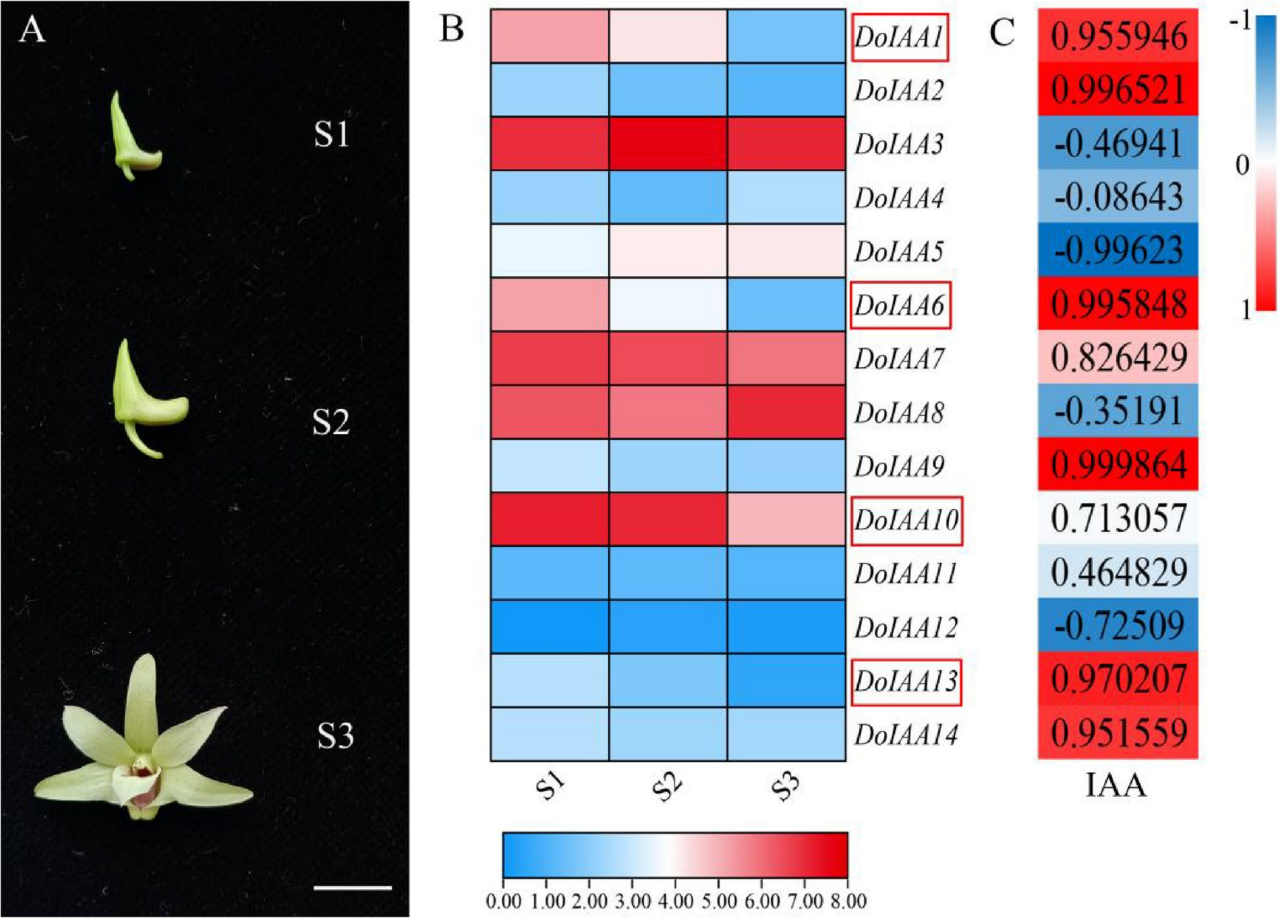
Expression patterns of 14 *DoIAA* genes at three flower development stages of *Dendrobium officinale*. **A** Three flower development stages. S1 indicates early flower buds, S2 indicates middle-stage flower buds, and S3 indicates fully-opened flowers. **B** Transcript profiles of *DoIAA* genes and the correlation coefficient with IAA content at the three flower developmental stages. Red represents a high transcript level and a positive correlation whereas blue represents a low transcript level and a negative correlation. Four genes (*DoIAA1*, *6*, *10*, *13*), indicated within red boxes, were used for the subcellular localization and yeast two-hybrid analysis

The potential roles of the 14 *Aux/IAA* genes at three stages of *D. officinale* flower development (S1, S2, S3) were assessed using RNA-seq data. Endogenous IAA content showed a decreasing trend from S1 to S3 [20], with most of the 14 *Aux/IAA* genes (*DoIAA1*, *2*, *6*, *7*, *9*, *10*, *13*, *14*) exhibiting a decreasing trend and a positive correlation (*r* > 0.7, *p* < 0.05) with IAA content, suggesting their important roles in IAA metabolism. Two *Aux/IAA* genes (*DoIAA5*, *12*) displayed an increasing trend from S1 to S3, and were negatively correlated (*r* < -0.5, *p* < 0.05) with IAA content. Four other *Aux/IAA* genes (*DoIAA3*, *4*, *8*, *11*) exhibited unchanged or alternative trends (decreasing from S1 to S2, increasing from S2 to S3, peaking at S2) with IAA content (-0.5 < *r* < 0.7) (Fig. 4).

The relative expression levels of *DoARF* genes were assessed at S1-S3. Unlike the *Aux/IAA* genes, the 26 *DoARF* genes exhibited different expression patterns. Eight genes (*DoARF3*, *4*, *5*,* 6*,* 9*, *11*, *19*, *20*) showed an increasing trend from S1-S3, and were negatively correlated (*r* < -0.5, *p* < 0.05) with IAA content. In contrast, nine genes (*DoARF1*, *2*, *10*, *16*, *17*, *18*, *21*, *22*, *23*) displayed a decreasing trend from S1-S3 and exhibited a positive correlation (*r* > 0.7, *p* < 0.05) with IAA content. Another nine genes (*DoARF7*, *8*, *12*, *13*, *14*, *15*, *24*, *25*, *26*) exhibited an unchanged or alternative trend (increasing from S1 to S2, decreasing from S2 to S3, peaking at S2) with IAA content (-0.5 < *r* < 0.7) (Fig. S2).

**Fig. 5:**
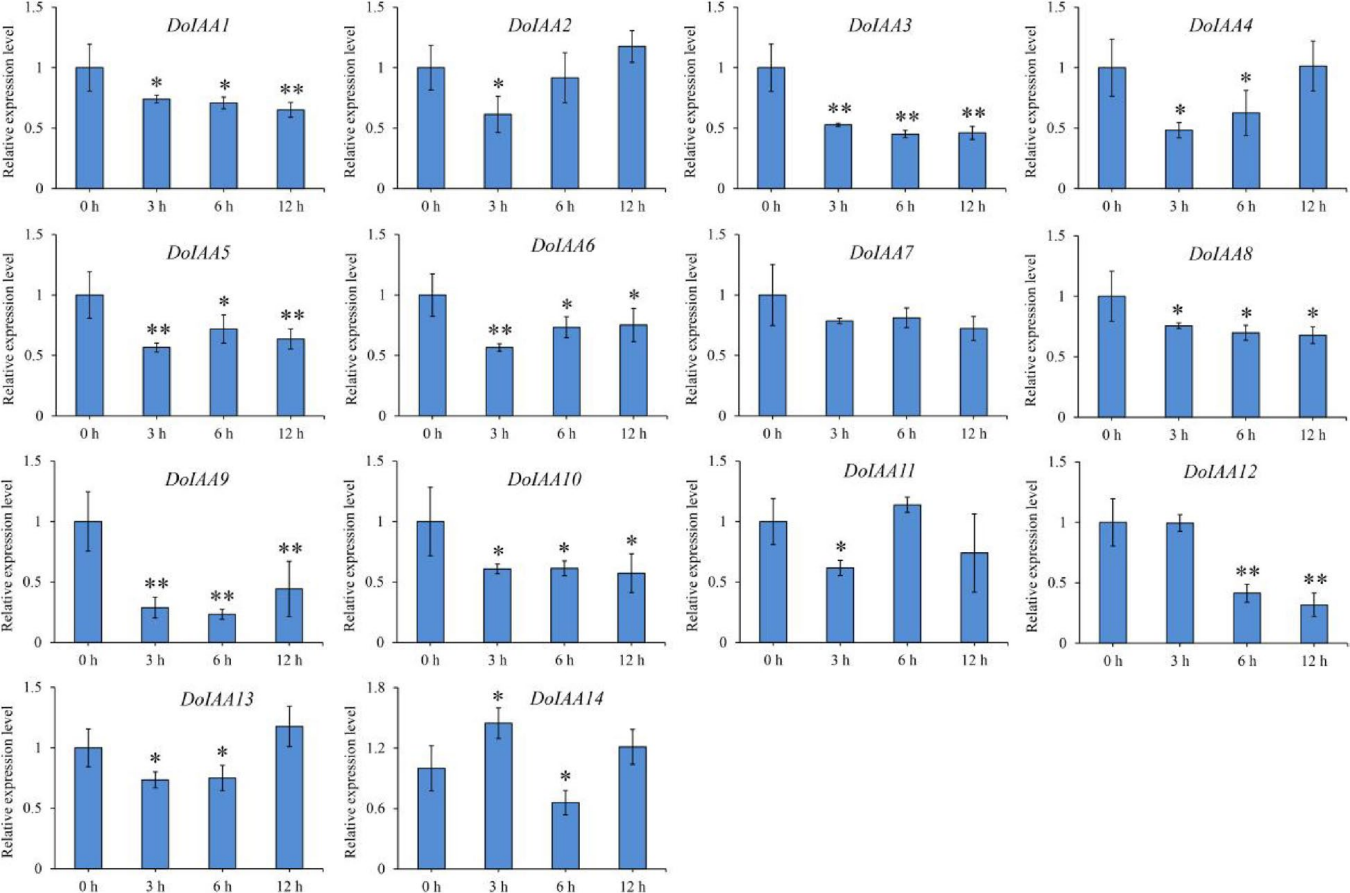
Expression patterns of 14 *DoIAA* genes in the IAA treatment. Error bars represent the mean ± standard deviation (SD) of three biological replicates (*n* = 3). * and ** indicate significant differences at *p* < 0.05 and *p* < 0.01, respectively (DMRT)

To further understand the possible roles of *Aux/IAA* genes in the IAA signaling pathway, 10-month-old tissue-cultured seedlings were exposed to exogenous IAA. In the IAA treatment, nearly all of the 14 *Aux/IAA* genes (except for *DoIAA7*) were down-regulated in leaves. Among them, four *Aux/IAA* genes (*DoIAA3*,* 4*, *9*, *12*) displayed two-fold lower expression than the control (0 h) (Fig. 5). It is possible that exogenous IAA may inhibit the biosynthesis of endogenous IAA. Consequently, in such a case, the genes that participate in the metabolism of endogenous IAA would exhibit a decreasing trend.

### Localization of four Aux/IAA proteins

**Fig. 6:**
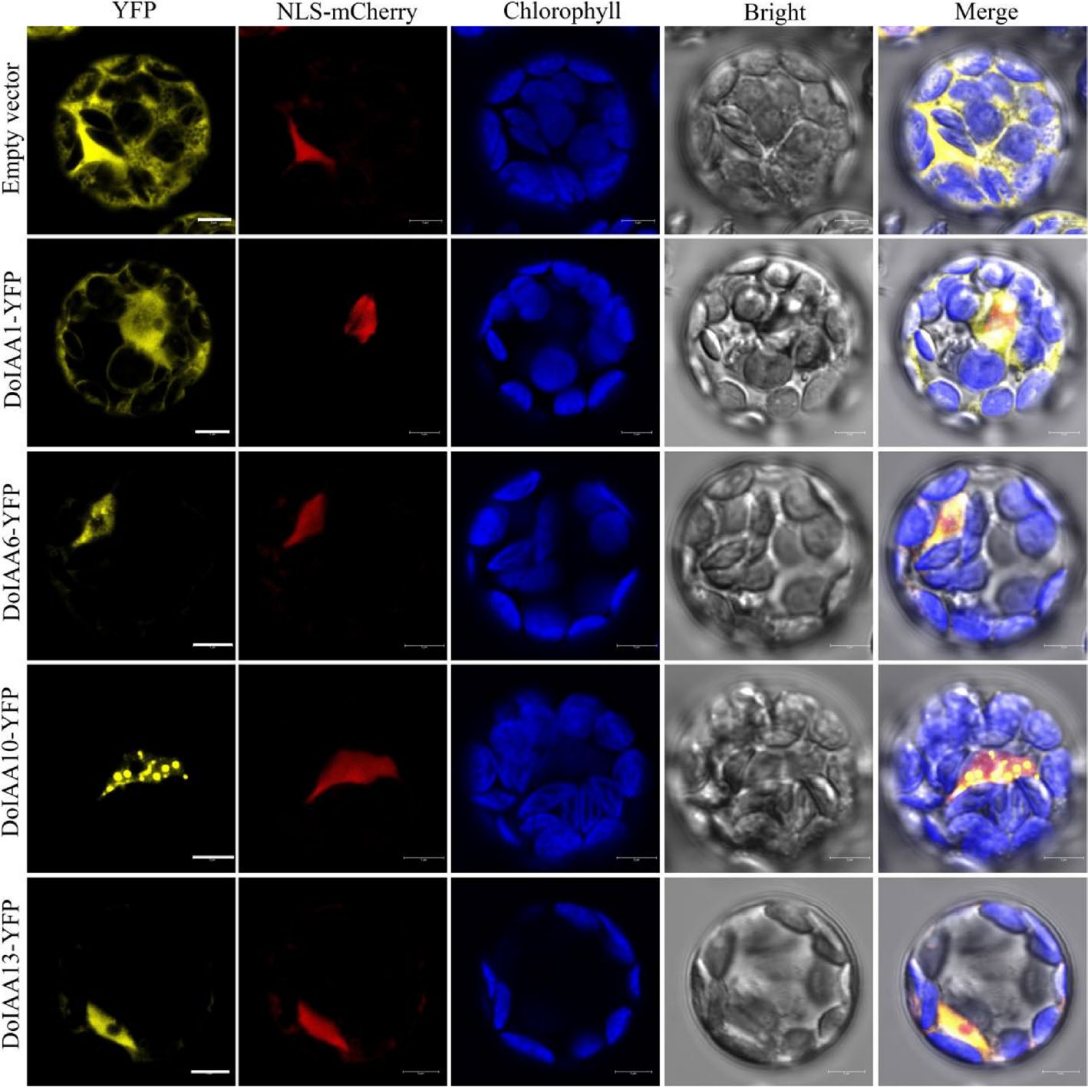
Subcellular localization of YFP, DoIAA1-YFP, DoIAA6-YFP, DoIAA10-YFP and DoIAA13-YFP with nucleus localization marker NLS mCherry. Bars = 5 μm

To verify their predicted localization, four *DoIAA* genes (*DoIAA1*, *6*, *10* and *13*), which showed a positive correlation (*r* > 0.7, *p* < 0.05) with IAA content at the three stages of flower development, were selected to analyze subcellular localization. Yellow fluorescent signals of protoplasts transformed with DoIAA1-YFP were mainly localized in the nucleus and cytosol, while the three remaining fusion vectors were co-localized with NLS-mCherry and were primarily localized in the nucleus (Fig. 6). These results indicate that these four DoIAAs were localized in the nucleus but may have a protein–protein interaction with other proteins, such as DoARFs.

### Interaction analysis of DoIAA and DoARF proteins

**Fig. 7:**
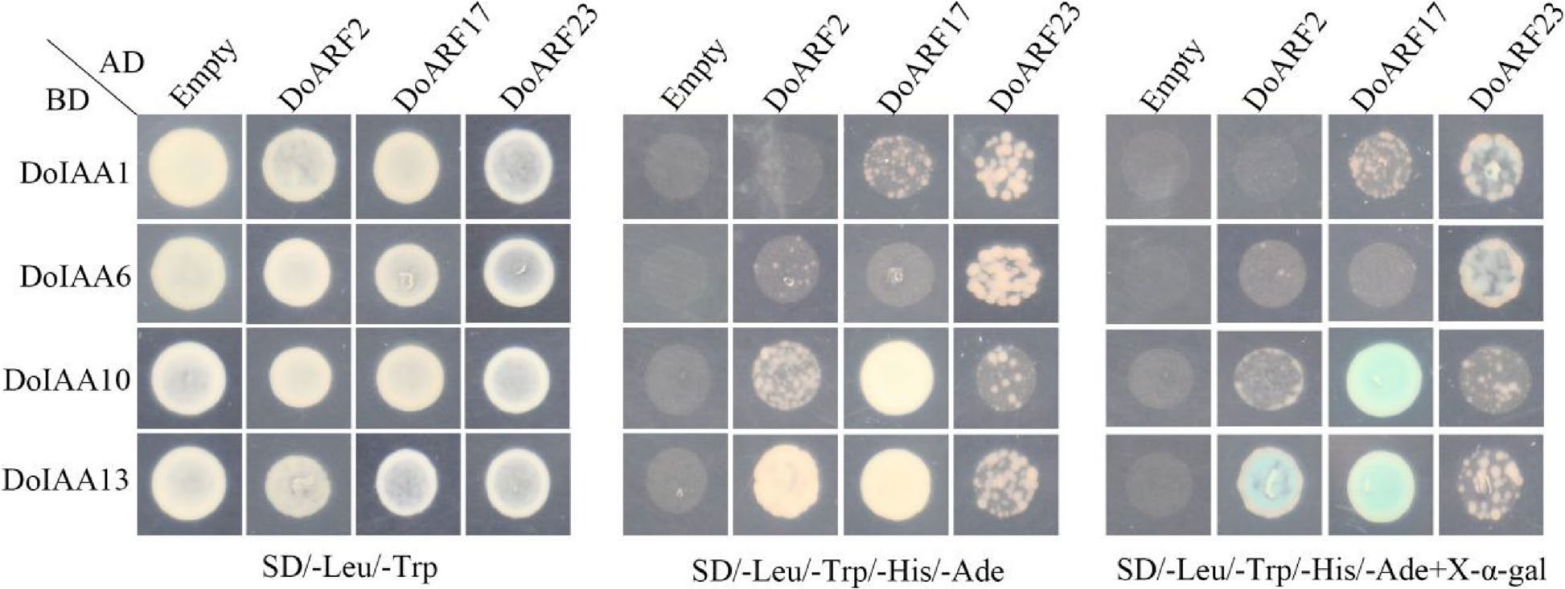
Analysis of the interaction between Aux/IAA and ARF proteins using a yeast two-hybrid assay. DoARF2, 17 and 23 were cloned into the pGADT7 vector; DoIAA1, 6, 10 and 13 were cloned into the pGBKT7 vector. All recombinants were transformed into yeast strain AH109 and plated on the following double or quadruple selection media: SD/-Leu/-Trp, SD/-Leu/-Trp/-His/-Ade and SD/-Leu/-Trp/-His/-Ade + X-α-gal. An empty vector was used as the control

To investigate the protein–protein interaction network of Aux/IAA and ARF proteins in the IAA signaling pathway of *D. officinale*, a yeast two-hybrid (Y2H) assay was conducted. Three *ARF* genes (*DoARF2*, *17*, *23*) and four *Aux/IAA* genes (*DoIAA1*, *6*, *10*, *13*), which displayed a positive correlation (*r* > 0.7, *p* < 0.05) with IAA content at three stages of flower development, were selected for a protein–protein interaction analysis. The negative control, containing pGADT7 (AD)-empty plus pGBKT7 (BD)-DoIAA1, BD-DoIAA6, BD-DoIAA10, BD-DoIAA13, grew well on double selection medium (SD/-Leu/-Trp) but did not survive on quadruple selection medium (SD/-Leu/-Trp/-His/-Ade). Transformed yeast cells containing AD-DoARF2 plus BD-DoIAA13, AD-DoARF17 plus BD-DoIAA10 and BD-DoIAA13, and AD-DoARF23 plus BD-DoIAA1 and BD-DoIAA6, grew well on double and quadruple selection medium, and subsequently produced blue colonies on medium supplemented with X-α-gal. Other recombinant bait carriers grew well on double selection medium but could not grow on quadruple selection medium, consistent with the results of the negative control (Fig. 7). The results of the Y2H assay indicate that DoARF2/17 directly interacted with DoIAA10/13.

## Discussion

To meet the market demand for human needs, wild *D. officinale* resources have been over-exploited and currently face extinction [21]. Plant tissue culture is able to alleviate this pressure on natural populations, allowing the artificial mass propagation of this plant [22]. Despite a wealth of studies on the tissue culture of *D. offcinale*, in order to meet market demands pertaining to both quality and volume, a better understanding of the ideal tissue culture factors [23], as well as the molecular biology [24] of growth and developmental processes, is needed. This paper adds to the body of knowledge related to growth and development in this orchid by advancing new information related to auxin biosynthesis.

The genome-wide identification of the *Aux/IAA* gene family has been achieved in many monocotyledonous and dicotyledonous plant species, including *Bletilla striata* (27 members) [25], *Dendrocalamus sinicus* (26 members) [26], *Hordeum vulgare* (36 members) [27], *Trichosanthes dioica* (37 members) [28], *Acer rubrum* (17 members) [29] and *Ricinus communis* (19 members) [30]. In this study, we reported the first systematic analysis of the *Aux/IAA* and *ARF* gene family in the *Dendrobium* genus, and a total of 14 putative *Aux/IAA* and 26 *ARF* genes were validated from the *D. officinale* genome*.* The number of *Aux/IAA* genes that we identified in *D. officinale* was similar to the number documented for *A. rubrum* and *R. communis*, but less than that noted for the other plant species indicated above based on a genomic analysis. This might indicate that a large-scale duplication event occurred late in the evolution of *D. officinale*, *A. rubrum* and *R. communis*, relative to other plants [31].

Similar to prior discoveries in another *Dendrocalamus* species [26], the 14 DoIAA proteins could be classified into two major groups, indicating that there are conserved motifs of Aux/IAA proteins in both *D. officinale* and *D. sinicus*. The conserved domain analysis revealed that all of the DoIAA proteins contained domains III and IV whereas a few did not include domains I or II, consistent with the Aux/IAA proteins of *A. thaliana* and *O. sativa* [32, 33]. The leucine-rich domain I and the proline-rich domain II acted as transcriptional repressors and were shown to be responsible for the characteristic instability of IAA proteins [34]. In our study, the stability of DoIAA5, 9, 12, 13 and 14, which lack domain II, may be higher than that of other proteins (Fig. 2). The IAA proteins, which have a bipartite NLS and SV40-type NLS in domains II and IV, tend to be localized in the nucleus. Consistent with this statement, four DoIAA proteins (DoIAA1, 6, 10 and 13) were localized in the nucleus (Fig. 6).

In *D. officinale*, quantitative real time polymerase chain reaction (qRT-PCR) data was strongly and positively correlated with RNA-seq data [18]. This technique is thus a useful and convenient tool for reflecting the transcript levels of target genes in different organs, developmental stages and phytohormone treatments. In pepper, *Aux/IAA* genes showed organ-specific expression, displaying different expression profiles in various organs [35]. The *DoIAA* genes also exhibited tissue-specific transcriptional patterns, indicating their important roles in root or flower development (Fig. 3). Concomitantly, endogenous IAA content decreased from S1-S3 and the expression levels of *DoIAA* genes displayed a similar trend as IAA content (Fig. 4), indicating their vital functions in the IAA signaling pathway.

*Aux/IAA* genes are thought to be primary early auxin-responsive genes, and their expression patterns vary depending on the plant species when treated with IAA, IAA analogues or other auxins. For example, in *O. sativa*, most *Aux/IAA* genes were upregulated by treatment with 2,4-dichlorophenoxy-acetic acid [33]. In *H. vulgare*, the *Aux/IAA* genes displayed various expression patterns when plants were sprayed with 1-naphthaleneacetic acid [27]. In *A. rubrum*, *Aux/IAA* genes exhibited positive responses or no changes to IAA treatment in new leaves [29]. In our research, most *Aux/IAA* genes were downregulated by IAA treatment (Fig. 5). We speculate that when plants were sprayed with exogenous IAA, the biosynthesis of the endogenous IAA was inhibited. DoIAA proteins form dimers with DoARFs, thereby repressing the auxin-responsive genes (e.g., *DoIAA*s) [36].

Domains III and IV of the C-terminus mediated the ARF-Aux/IAA interaction in *A. thaliana*, as validated by Y2H and biomolecular fluorescence complementation assays [36, 37]. In *Populus tomentosa*, PtoIAA9 interacted with PtoARF5 homologs via the CTD domain and regulated the expression of genes associated with early xylem development [38]. In apple (*Malus domestica*), MdARF13 interacted with an unstable Aux/IAA repressor MdIAA121 and regulated anthocyanin biosynthesis through the Aux/IAA-ARF signaling pathway [39]. In our study, DoIAA10 and 13 interacted with DoARF2 and 17, as shown by the Y2H assay (Fig. 7), and the expression levels of the four corresponding genes were downregulated during flower senescence (Fig. 4 and S2), indicating that they were involved in the auxin-signaling pathway of flower development.

**Fig. 8:**
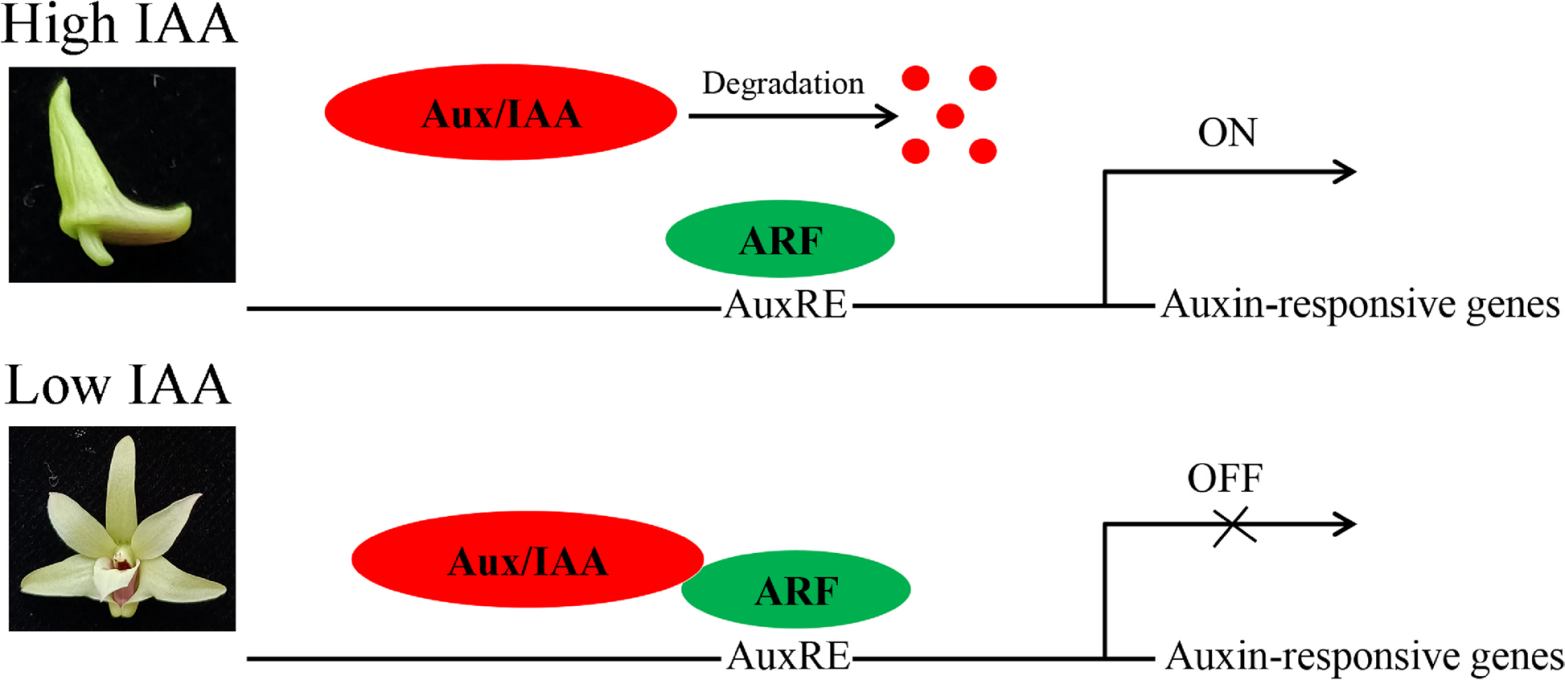
A proposed model of the Aux/IAA-ARF module in the mediation of auxin-signaling during *Dendrobium officinale* flower development

Based on this information, we propose a model: At the S1/S2 stage of flower development (flower buds with a high IAA concentration), Aux/IAA proteins are ubiquitinated and degraded by 26S proteasome [40]. ARF proteins are then released and activate the transcription of auxin-responsive genes. In S3 (fully-opened flowers with a low IAA concentration), Aux/IAA proteins interact with ARF proteins and repress the transcription of auxin-responsive genes [41]. Detailed biochemical and physiological experiments would be needed to confirm, or disprove, this proposed model (Fig. 8).

## Conclusion

In this study, the early auxin-responsive genes, including 14 *Aux/IAA* and 26 *ARF* genes, were validated from *D. officinale*, an orchid. By analyzing sequence homologues, it was found that most of the Aux/IAA proteins contained four conserved domains (I, II, III, and IV) and two NLSs. In different organs, the *Aux/IAA* genes displayed tissue-specific patterns, indicating their important roles in root/flower development. In the IAA treatment, most of the *Aux/IAA* genes were downregulated. Four *Aux/IAA* genes (*DoIAA1*, *6*, *10*, *13*) and three *ARF* genes (*DoARF2*, *17*, *23*), which were positively correlated with IAA content during flower development, were selected for further analysis. As shown by a Y2H assay, two Aux/IAA proteins (DoIAA10, 13) displayed a protein–protein interaction with ARF proteins (DoARF2, 17), suggesting that the IAA may regulate flower development in *D. officinale* via an Aux/IAA-ARF–mediated signaling pathway.

## Methods

### Plant materials, growth conditions and hormone treatments

Tissue-cultured *D. officinale* cultivar ‘Zhongke 5’ was collected from Liancheng of Fujian province in China and cultivated by professor Jun Duan (https://www.cas.cn/syky/201811/t20181109_4669776.shtml). This research complies with the IUCN Policy Statement on Research Involving Species at Risk of Extinction and the Convention on the Trade in Endangered Species of Wild Fauna and Flora. Seedlings were cultured at the South China Botanical Garden, Chinese Academy of Sciences, Guangzhou, in China. They were planted in vitro in jars (about 10 seedlings in 100 mL medium of 600 mL volume per jar) with Murashige and Skoog (MS) medium containing 0.5% (w/v) activated carbon under controlled environmental conditions: 24 ± 2 °C; 12-h photoperiod (80 μmol/m^2^·s); 80% relative humidity [42].

Ten-month-old tissue-cultured seedlings were divided into four groups with three biological replicates each. Whole plants in jars were sprayed (volume per jar) with 20 mL of 10 μmol/L IAA (Yuanye Biotechnology Co., Ltd., Shanghai, China) for different periods of time (0, 3, 6, and 12 h). Separately, plants that were not sprayed with IAA (0 h) were used as the control. For each treatment, young leaves near the shoot tip were collected and mixed, frozen in liquid nitrogen and stored at -80 °C for RNA extraction.

To analyze subcellular localization, *A. thaliana* (ecotype Columbia) seeds (*v* = 50 μL) were sprayed with tap water and stored in the dark at 4 °C for 48 h. Seeds were then transferred to a substrate containing nutrient soil and vermiculite (*v*:* v* = 2:1), and placed in a controlled growth environment (22 ± 2 °C; 16-h photoperiod; 80 μmol/m^2^·s; 80% relative humidity). When *A. thaliana* plants were 4–6 weeks old, protoplasts were harvested from leaves using a previously described method [43].

### Bioinformatics of the *Aux/IAA* and *ARF* gene family

The Hidden Markov Model (HMM) of the *Aux/IAA* gene family (PF02309) and *ARF* gene family (PF06507) were generated from the pfam database (http://pfam-legacy.xfam.org/) and used to find putative *Aux/IAA* and *ARF* genes in the *D. officinale* genome [44] using the hmmbuild tool (http://www.hmmer.org/). The promoter regions (about -1500 bp before ATG) of *Aux/IAA* genes were selected from the scaffold data in the *D. officinale* genome [44] and used to analyze candidate CREs in PlantCARE software (http://bioinformatics.psb.ugent.be/webtools/plantcare/html/). The amino acid sequences of putative Aux/IAA proteins were submitted to Expasy (https://www.expasy.org/) to calculate the number of AAs, pI and MW, and also submitted to WoLF PSORT (https://wolfpsort.hgc.jp/) to predict subcellular localization. The Aux/IAA proteins from *D. officinale* were aligned by ClustalX (http://www.clustal.org/) and submitted to the DNAMAN software (https://www.lynnon.com/index.html) to conduct an analysis of multiple sequence alignment. The Aux/IAA proteins from *D. officinale*, *A. thaliana* and *Oryza sativa* [28, 29] were aligned by default parameters of Muscle built in MEGA-X software (https://www.megasoftware.net/), and used to construct a neighbor-joining (N-J) tree with MEGA-X software (https://www.megasoftware.net/).

### Subcellular localization of Aux/IAA proteins

To construct YFP-fusion vectors, the ORFs of the *DoIAA* genes without the stop codon were cloned into the *Nco*I site of pSAT6-EYFP-N1 [45]. The empty plasmid and recombinant plasmids with NLS-mCherry localization marker, which are located in the nucleus, were co-transferred into the protoplasts of *A. thaliana* using polyethylene glycol-mediated transformation [43]. After incubation at 22 °C in the dark for 12–16 h, the YFP fluorescence signals of protoplasts were visualized under a Leica TCS SP8 STED 3 × microscope (Leica Camera AG., Solms, Germany). All of the primers designed for subcellular localization are listed in Table S2.

### Y2H assay

The *Aux/IAA* and *ARF* genes that displayed a decreasing trend during flower development were selected for the Y2H assay. To construct AD/BD recombinant vectors, the ORFs of four *Aux/IAA* genes (*DoIAA1*, *6*, *10* and *13*) were cloned into the pGBKT7 vector (Clontech, Palo Alto, CA, USA), and the ORFs of three *ARF* genes (*DoARF2*, *17* and *23*) were cloned into the pGADT7 vector (Clontech). Two vectors (containing DoIAAs-BD and DoARFs-AD) were co-transformed into yeast strain AH109-competent cells (Weidi Biotech Co Ltd., Shanghai, China) following the manufacturer’s instructions and plated onto double (SD/-Leu/-Trp) and quadruple (SD/-Leu/-Trp/-His/-Ade) selection medium. Media were maintained at 30 °C for 2–4 days. 5-Bromo-4-chloro-3-indolyl-α-D-galactoside (X-α-gal) (Coolaber, Beijing, China) was added to the SD/-Leu/-Trp/-His/-Ade medium to test for β-galactosidase (LacZ) activity [46]. All the primers used in the Y2H assay are listed in Table S2.

### Transcriptomic analysis

The RNA-seq data of different tissues, including of the column, sepals, white part of roots, green root tips, stems, leaves, lip and flower buds [47], is available at the SRA database (https://www.ncbi.nlm.nih.gov/sra/) under Bioproject number PRJNA348403. The RNA-seq data of three developmental stages of flowers, including S1 (early flower buds), S2 (medium-stage flower buds) and S3 (fully-opened flowers) [20], were downloaded from the National Genomics Data Center (https://ngdc.cncb.ac.cn/) under BioProject number PRJCA003343. Raw sequences were transformed into clean reads and mapped to the *D. officinale* genome [44]. The fragments per kilobase of transcript per million fragments mapped (FPKM) value was used to calculate the levels of transcription of *Aux/IAA* and *ARF* genes. Heatmaps of the level of expression of *DoIAA* genes were visualized in TBtools [48].

### RNA extraction, cDNA synthesis and qRT-PCR

The RNA of leaves was extracted and purified (by removing DNA) by the Quick RNA Isolation Kit (0416–50 GK, Huayueyang Biotechnology Co. Ltd., Beijing, China). RNA (1 μg) of different samples from the IAA treatment were reverse transcribed to cDNA by the GoScript™ Reverse Transcription System (Promega, Madison, WI, USA) according to the manufacturer’s instructions. cDNA was diluted to 300 ng/μL with double distilled water and used as a template for qRT-PCR. The qRT-PCR assay with the Unique Aptamer™ qPCR SYBR® Green Master Mix (Novogene, Tianjin, China) was performed using the LightCycler® 480 II real-time PCR system (Roche, Basel, Switzerland). The cycling parameters that were used are as follows: a hot start (95 °C) for 2 min; 40 cycles of [95 °C for 15 s, 60 °C for 1 min and 95 °C for 15 s]; 60 °C for 1 min; 95 °C for 15 s; 60 °C for 15 s. The expression levels of target genes were quantified by the 2^−ΔΔCT^ method [49] using *EF-1α* [50] and *Actin* (NCBI No. JX294908) as the reference genes. All primers were designed by Integrated DNA Technologies (https://sg.idtdna.com/PrimerQuest/Home/) and are listed in Table S3. Three independent biological and technical replicates were conducted.

### Statistical analysis

Data were analyzed by Sigmaplot 12.0 (Systat Software Inc., San Jose, CA, USA). After one-way analysis of variance between the means of different mRNA levels of *Aux/IAA* and *ARF* genes in the IAA treatment, significance was assessed by Duncan’s multiple range test (DMRT) at *p* < 0.05 or *p* < 0.01. Correlation analysis between the transcriptional levels of *Aux/IAA* and *ARF* genes and IAA concentration during flower development was performed using Pearson’s correlation coefficient (*r*) at *p* < 0.05.

## Supplementary Information

**Additional file 1:** **Fig. S1.** Prediction of *cis*-regulatory elements (CREs) in the promoter regions of *DoIAA* genes. Different colors represent different CREs. **Fig. S2.** Expression patterns of 26 *DoARF* genes at three flower developmental stages of *Dendrobium officinale*. S1, early flower buds; S2, middle-stage flower buds; S3, fully-opened flowers. Transcript profiles of *DoARF* genes and the correlation coefficient with IAA content in S1-S3. Red represents a high transcript level and positive correlation, and blue represents a low transcript level and negative correlation. Three genes (*DoARF2*, *17*, *23*), indicated by red boxes, were used for the yeast two-hybrid analysis. **Table S1.** Physiochemical parameters of the 26 DoARF proteins. **Table S2.** Primers designed for subcellular localization and yeast two-hybrid assay. **Table S3.** Primers designed for qRT-PCR assay.

## Data Availability

Raw RNA-seq data of different tissues is available at the NCBI under BioProject number PRJNA348403 (https://www.ncbi.nlm.nih.gov/bioproject/PRJNA348403). Raw RNA-seq data of flower development is available at the NGDC under BioProject number PRJCA003343 (https://ngdc.cncb.ac.cn/search/?dbId=&q=PRJCA003343).

## Abbreviations

ABA: Abscisic acid
Aux: Auxin
AuxRE: Auxin response element
ARF: Auxin response factor
CRE: *cis*-Regulatory element
CTD: C-terminal protein–protein interaction domain
DBD: DNA-binding domain
DMRT: Duncan’s multiple range test
FPKM: Fragments per kilobase of transcript per million fragments mapped
GA: Gibberellin acid
GH3: Auxin-responsive gretchen hagen3
HMM: Hidden Markov model
IAA: Indole-3-acetic acid
pI: Isoelectric point
MAP: Medicinal and aromatic plant
MeJA: Methyl jasmonate
MW: Molecular weight
MS: Murashige and Skoog
NLS: Nuclear localization signal
ORF: Open reading frame
N-J: Neighbor-joining
PEG: Polyethylene glycol
qRT-PCR: Quantitative real time polymerase chain reaction
SA: Salicylic acid
SAUR: Small auxin upregulated RNA
SD: Standard deviation
SM: Secondary metabolite
X-α-gal: 5-Bromo-4-chloro-3-indolyl-α-D-galactoside
YFP: Yellow fluorescent protein
Y2H: Yeast two-hybrid
