# Supplementary material for: Genome-wide identification of Aux/IAA and ARF gene families reveal their potential roles in flower opening of Dendrobium officinale
Source: BMC Genomics. 2023 Apr 13;24:199. doi: 10.1186/s12864-023-09263-y (PMC10099678; doi:10.1186/s12864-023-09263-y)
Supplement: Supplementary file 1 — Additional file 1: Fig. S1. Prediction of cis-regulatory elements (CREs) in the promoter regions of DoIAA genes. Different colors represent different CREs. Fig. S2. Expression patterns of 26 DoARF genes at three flower developmental stages of Dendrobium officinale. S1, early flower buds; S2, middle-stage flower buds; S3, fully-opened flowers. Transcript profiles of DoARF genes and the correlation coefficient with IAA content in S1-S3. Red represents a high transcript level and positive correlation, and blue represents a low transcript level and negative correlation. Three genes (DoARF2, 17, 23), indicated by red boxes, were used for the yeast two-hybrid analysis. Table S1. Physiochemical parameters of the 26 DoARF proteins. Table S2. Primers designed for subcellular localization and yeast two-hybrid assay. Table S3. Primers designed for qRT-PCR assay. [file 12864_2023_9263_MOESM1_ESM.docx]

Supplementary figure and table legends

Fig. S1. Prediction of *cis*-regulatory elements (CREs) in the promoter regions of *DoIAA* genes. Different colors represent different CREs.

Fig. S2. Expression patterns of 26 *DoARF* genes at three flower developmental stages of *Dendrobium officinale*. S1, early flower buds; S2, middle-stage flower buds; S3, fully-opened flowers. Transcript profiles of *DoARF* genes and the correlation coefficient with IAA content in S1-S3. Red represents a high transcript level and positive correlation, and blue represents a low transcript level and negative correlation. Three genes (*DoARF2*, *17*, *23*), indicated by red boxes, were used for the yeast two-hybrid analysis.

Table S1. Physiochemical parameters of the 26 DoARF proteins.

Table S2. Primers designed for subcellular localization and yeast two-hybrid assay.

Table S3. Primers designed for qRT-PCR assay.

Fig. S1


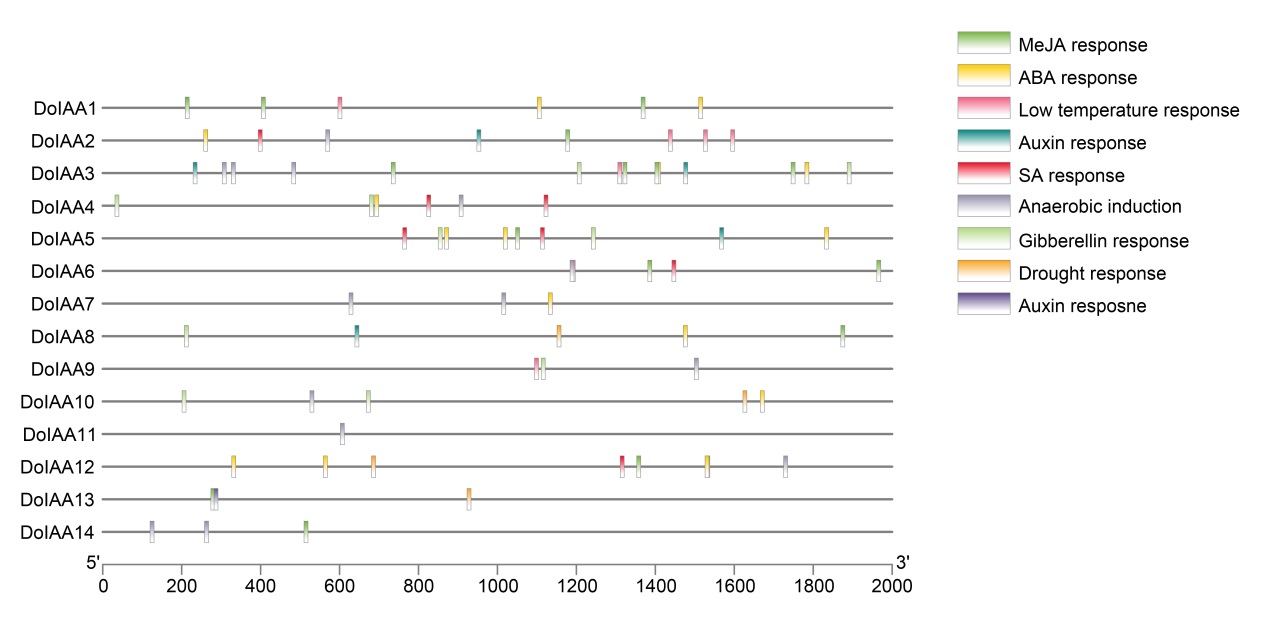


Fig. S2


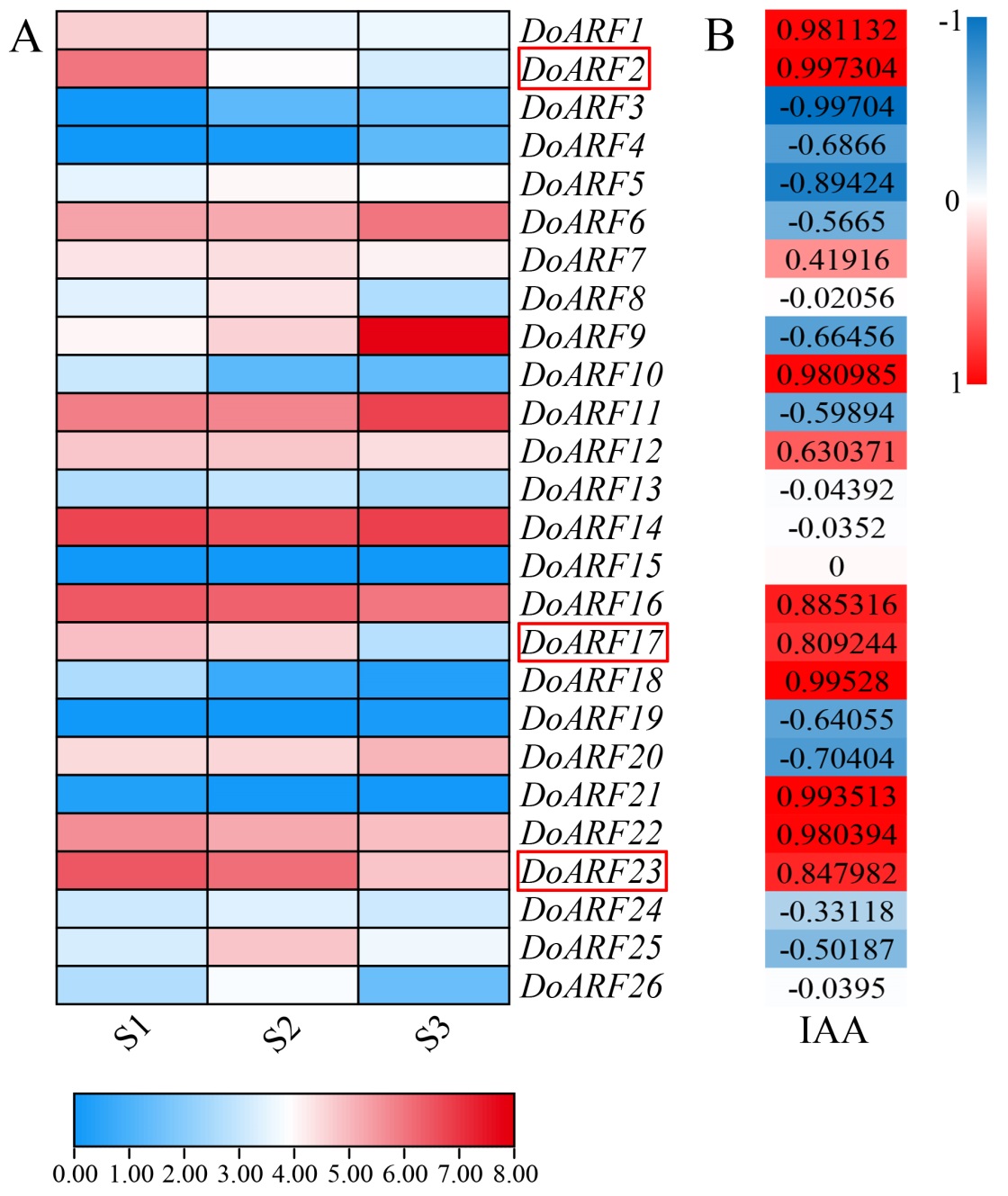


| Gene ID | Locus | ORF (bp) | AA (aa) | pI | Mw (KDa) | Localization |
| --- | --- | --- | --- | --- | --- | --- |
| *DoARF1* | Dca000054 | 2409 | 802 | 5.82 | 88.92 | Nucleus |
| *DoARF2* | Dca003692 | 2535 | 844 | 6.41 | 94.01 | Nucleus |
| *DoARF3* | Dca004596 | 231 | 76 | 4.69 | 8.33 | Cytoplasm |
| *DoARF4* | Dca004597 | 1158 | 385 | 6.18 | 43.41 | Nucleus |
| *DoARF5* | Dca004728 | 1842 | 613 | 7.21 | 68.45 | Nucleus |
| *DoARF6* | Dca005102 | 2469 | 822 | 5.66 | 89.83 | Nucleus |
| *DoARF7* | Dca005702 | 2061 | 686 | 7.27 | 77.50 | Nucleus |
| *DoARF8* | Dca009036 | 2106 | 701 | 8.06 | 77.63 | Peroxisome |
| *DoARF9* | Dca009918 | 3495 | 1164 | 6.26 | 129.40 | Nucleus |
| *DoARF10* | Dca011291 | 2658 | 885 | 5.03 | 98.65 | Nucleus |
| *DoARF11* | Dca011479 | 2100 | 699 | 6.71 | 77.63 | Nucleus |
| *DoARF12* | Dca011873 | 2427 | 808 | 6.00 | 90.77 | Nucleus |
| *DoARF13* | Dca012478 | 2820 | 939 | 6.25 | 105.96 | Nucleus |
| *DoARF14* | Dca013368 | 2730 | 909 | 5.98 | 101.16 | Nucleus |
| *DoARF15* | Dca014960 | 1713 | 570 | 4.59 | 64.18 | Cytoplasm |
| *DoARF16* | Dca015884 | 2310 | 769 | 5.75 | 86.22 | Nucleus |
| *DoARF17* | Dca019461 | 2643 | 880 | 6.40 | 98.55 | Nucleus |
| *DoARF18* | Dca020629 | 1995 | 664 | 6.70 | 74.50 | Nucleus |
| *DoARF19* | Dca020978 | 180 | 59 | 8.68 | 6.98 | Chloroplast |
| *DoARF20* | Dca023413 | 2019 | 672 | 5.84 | 74.83 | Nucleus |
| *DoARF21* | Dca023708 | 210 | 69 | 3.76 | 7.99 | —— |
| *DoARF22* | Dca024139 | 2532 | 843 | 6.55 | 93.70 | Nucleus |
| *DoARF23* | Dca025204 | 1986 | 661 | 5.62 | 73.87 | Nucleus |
| *DoARF24* | Dca025290 | 1527 | 508 | 9.09 | 57.63 | Chloroplast |
| *DoARF25* | Dca026139 | 2001 | 666 | 7.60 | 74.53 | Nucleus |
| *DoARF26* | Dca026323 | 1569 | 522 | 8.61 | 57.58 | Chloroplast |

Table S1

Table S2

| Gene | Type | Primer sequences (5′-3′) |
| --- | --- | --- |
| pSAT6-EYFP-N1-DoIAA construction | | |
| *DoIAA1* | F | CGAACGATAGCCATGGAGATGACGCCGCCGTTGGAGCAT |
|  | R | TGAGTCCGGACCATGGTCTCCCATGGAACATCACCGAC |
| *DoIAA6* | F | CGAACGATAGCCATGGAGATGGAAACAGAGTTTAGAAAG |
|  | R | TGAGTCCGGACCATGGTCTTCCAAGGAACATCCCCAAC |
| *DoIAA10* | F | CGAACGATAGCCATGGAGATGTCGCCGCCACTTGAACTC |
|  | R | TGAGTCCGGACCATGGTGTTTCGATTTTTGTTCTTCTC |
| *DoIAA13* | F | CGAACGATAGCCATGGAGATGCTGAGTTCACAGGCAACC |
|  | R | TGAGTCCGGACCATGGTACTCTCATATTCTTTAGCATC |
| pGBKT7-DoIAA construction | | |
| *DoIAA1* | F | CATGGAGGCCGAATTCATGACGCCGCCGTTGGAGCAT |
|  | R | GGATCCCCGGGAATTCCTCCCATGGAACATCACCGAC |
| *DoIAA6* | F | CATGGAGGCCGAATTCATGGAAACAGAGTTTAGAAAG |
|  | R | GGATCCCCGGGAATTCCTTCCAAGGAACATCCCCAAC |
| *DoIAA10* | F | CATGGAGGCCGAATTCATGTCGCCGCCACTTGAACTC |
|  | R | GGATCCCCGGGAATTCGTTTCGATTTTTGTTCTTCTC |
| *DoIAA13* | F | CATGGAGGCCGAATTCATGCTGAGTTCACAGGCAACC |
|  | R | GGATCCCCGGGAATTCACTCTCATATTCTTTAGCATC |
| pGADT7-DoARF construction | | |
| *DoARF2* | F | GGAGGCCAGTGAATTCATGTACATGGCTGCGTCGGAG |
|  | R | CACCCGGGTGGAATTCGCAGTTCTCTACATCAGTTAT |
| *DoARF17* | F | GGAGGCCAGTGAATTCATGGCGTCGTTCGACGTCTCC |
|  | R | CACCCGGGTGGAATTCTCGAATTTCATGGCTCTTAGA |
| *DoARF23* | F | GGAGGCCAGTGAATTCATGGCTCTTGCTCCTCCCACT |
|  | R | CACCCGGGTGGAATTCGTCATCAGAGGGGCCAATGGC |

F: forward; R: reverse

| Gene | Type | Primer sequences (5′-3′) |
| --- | --- | --- |
| qRT-PCR analysis | | |
| *DoIAA1* | F | TCAGAAGCTACCGCAAGAATAC |
|  | R | AGAGGCAACCAAGTCCAATC |
| *DoIAA2* | F | TGCACTTGCAGAGAGAGAAG |
|  | R | GGTAGCTATCATGAGCCAGTAAA |
| *DoIAA3* | F | GGTAGGTAACTCGCCAAACA |
|  | R | CATCCAGTCACCATCCTTATCC |
| *DoIAA4* | F | CTCAGATAAGGGCTGCTTCTAC |
|  | R | GAGCTTCCAGGTTCAGATTCA |
| *DoIAA5* | F | GACTGGATGCTAGTTGGAGATG |
|  | R | GGCTCTGGATTGACTGATTGA |
| *DoIAA6* | F | GGCCACCTTCTAATCTCTCTTC |
|  | R | GAGCCCAAGAATCCCACTTT |
| *DoIAA7* | F | TGAAGACAAGGATGGAGATTGG |
|  | R | GCGTCTGAGCCCTTCATAAT |
| *DoIAA8* | F | GTCGCAGGGCATACCTAATAAA |
|  | R | TCCAATCACCATCCTTGTCTTC |
| *DoIAA9* | F | CTACAAGCCCACCTCTTCTTT |
|  | R | TCTCCAACCAGCATCCAATC |
| *DoIAA10* | F | TTCCTGGTAGGGATGGACTAA |
|  | R | CGAGCATCCAATCTCCATCTT |
| *DoIAA11* | F | GCAGCTCTCATTCTCTCATCTG |
|  | R | CATCTCCCACCATCATCCAATC |
| *DoIAA12* | F | ACAGATGTTCAGGACCACAAT |
|  | R | TCTCCAACCATCATCCAATCTC |
| *DoIAA13* | F | TGGTTGACGAGGTGAAGAAC |
|  | R | GAGAGGGACTCATAGTTGGAATG |
| *DoIAA14* | F | GTTCTACAAACCCACCTCTTCT |
|  | R | ATCTCCAACCAACATCCAGTC |
| *DoActin* | F | TCCCAAGGCAAACAGAGAAA |
|  | R | GGCCACTAGCATATAGGGAAAG |
| *DoEF-1α* | F | TCAGGCTGACTGTGCTGTCCT |
|  | R | GTGGTGGCGTCCATCTTGTT |

Table S3

F: forward; R: reverse
